# A LysM and SH3-Domain Containing Region of the *Listeria monocytogenes* p60 Protein Stimulates Accessory Cells to Promote Activation of Host NK Cells

**DOI:** 10.1371/journal.ppat.1002368

**Published:** 2011-11-03

**Authors:** Rebecca L. Schmidt, Holly C. Filak, Jack D. Lemon, Terry A. Potter, Laurel L. Lenz

**Affiliations:** Integrated Department of Immunology, National Jewish Health and University of Colorado School of Medicine, Denver, Colorado, United States of America; University of Michigan Medical School, United States of America

## Abstract

*Listeria monocytogenes* (Lm) infection induces rapid and robust activation of host natural killer (NK) cells. Here we define a region of the abundantly secreted Lm endopeptidase, p60, that potently but indirectly stimulates NK cell activation *in vitro* and *in vivo*. Lm expression of p60 resulted in increased IFNγ production by naïve NK cells co-cultured with treated dendritic cells (DCs). Moreover, recombinant p60 protein stimulated activation of naive NK cells when co-cultured with TLR or cytokine primed DCs in the absence of Lm. Intact p60 protein weakly digested bacterial peptidoglycan (PGN), but neither muropeptide recognition by RIP2 nor the catalytic activity of p60 was required for NK cell activation. Rather, the immune stimulating activity mapped to an N-terminal region of p60, termed L1S. Treatment of DCs with a recombinant L1S polypeptide stimulated them to activate naïve NK cells in a cell culture model. Further, L1S treatment activated NK cells *in vivo* and increased host resistance to infection with *Francisella tularensis* live vaccine strain (LVS). These studies demonstrate an immune stimulating function for a bacterial LysM domain-containing polypeptide and suggest that recombinant versions of L1S or other p60 derivatives can be used to promote NK cell activation in therapeutic contexts.

## Author Summary

Pathogens have evolved diverse strategies to influence host immune responses. By studying these strategies, we may learn how to therapeutically intervene to manipulate immune responses during infectious and other diseases. In this study, we investigated how the bacterial pathogen *Listeria monocytogenes* (Lm) stimulates activation of an innate immune cell type called the natural killer (NK) cell. NK cells protect against certain infections, tumors, and autoimmune diseases, but appear to play a deleterious role in the context of Lm infection. We found that putative carbohydrate and protein interaction domains of a heavily secreted Lm protein and virulence factor, p60, indirectly stimulate NK cells both during infection and in the absence of other bacterial factors. Treatment of mice with this region of p60 stimulated NK cell activity and was protective in a mouse model of systemic infection by an NK cell sensitive bacterial pathogen, *Francisella tularensis*. These studies suggest that derivatives of p60 protein may prove to be useful tools for activation of NK cells and demonstrate therapeutic use of this bacterial immune modulating factor.

## Introduction

NK cells are lymphocytes that help control infections and tumors and regulate autoimmune responses through both cytotoxic and cytokine-secreting effector functions. Through the use of Fas, TRAIL, and secretion of perforin and granzyme B, NK cells induce apoptosis or lysis of distressed somatic cells. NK cells can also activate or suppress other aspects of an immune response through lysis of antigen-presenting cells or production of immune regulatory cytokines such as IFNγ and IL-10 [1], [2], [3].

NK cells are activated by secreted cytokines and by proteins on the surface of target cells or other immune cells. NK cells recognize “self” class I major histocompatibility complex (MHCI) cell surface molecules by inhibitory receptors that prevent NK cell activation [4]. Reduced expression of MHCI by stressed or infected target cells can thus relieve this inhibition and lead to NK cell activation. Alternatively, NK cells can be directly activated by recognition of stress-induced ligands through a variety of activating NK cell receptors [5]. In addition to direct recognition of target cells, the activity of NK cells is regulated by cytokines released from macrophages and dendritic cells, such as TNFα, IFNγ, IL-15, IL-12, and IL-18 [6], [7]. IL-18 is a potent stimulus for IFNγ production by (NK) cells [8], [9] Full NK cell activation often requires direct contact of the NK cell with accessory cells, such as dendritic cells (DCs). In some cases, contact of NK cells and DCs permits signaling of co-stimulatory molecules [6], [10], [11], [12]. Contact may also promote efficient transmission of IL-18 and/or IL-12 [13], [14], and *trans*-presentation of IL-15 to “prime” the NK cell [15], [16]. The balance of both inhibitory and activating signals ultimately determines the extent of NK cell activation and possibly the nature of NK cell effector functions.

Systemic infection by numerous bacterial pathogens elicits potent NK cell activation and IFNγ production, but the mechanisms of NK cell activation during bacterial infections are incompletely understood. Infection by *Listeria monocytogenes* (Lm) rapidly activates a large population of NK cells to produce IFNγ [17], [18]. Lm is a facultative intracellular pathogen of humans and animals [19]. A number of secreted Lm virulence factors that contribute to pathogenicity. One of the two most abundantly secreted Lm proteins is a bacterial hemolysin (Hly) called listeriolysin O. Hly is essential for bacterial access to the cytosol of host cells and thus for intracellular bacterial growth and virulence during systemic infection of mice [19], [20]. The second most heavily secreted Lm protein is called p60. Expression of p60 also contributes to Lm virulence during systemic infections [17], [21], [22]. However, the virulence-promoting function of p60 has been enigmatic. The p60 sequence contains a C-terminal NLPC/p60 domain, two N-terminal LysM domains, and a single N-terminal SH3-like domain. Some NLPC/p60 domains have been associated with endopeptidase activity [23], [24], while LysM and bacterial SH3 domains generally bind glycans or proteins [25], [26], [27], [28]. Consistent with autolytic endopeptidase activity, semi-purified p60 protein digested *Micrococcus luteus* cells [29], [30], and crude Lm PGN [17]. We previously hypothesized that Lm expression of p60 might thus contribute to Lm pathogenicity by altering the production of immune modulating muropeptides [21]. Subsequently, an immune modulatory function was associated with Lm expression of p60. Namely, systemic infections by wt Lm promoted significantly increased NK cell activation when compared to infections by p60-deficient (Δp60) Lm [17].

Here, we confirm that p60 deficiency correlates with impaired NK cell activation in a recently developed cell culture assay system. Furthermore, using recombinant p60 protein and p60-derived polypeptides, we show that p60 protein can indirectly enhance NK cell activation in the absence of additional Lm factors. Purified p60 protein binds to DCs and induces IL-18 secretion, which is required for NK cell activation by p60 in co-culture. The ability of p60 to stimulate DCs for NK cell activation mapped to the first LysM and SH3 domains (L1S) of the p60 protein. The L1S region was also sufficient to promote activation of NK cells *in vivo* when given to naïve mice. *In vivo* treatment with p60 increased serum IFNγ and reduced susceptibility of recipient mice to infection by the heterologous NK cell-sensitive bacterial pathogen, *Francisella tularensis*. These data demonstrate that p60 protein boosts NK cell activation during Lm infection through appropriate stimulation of accessory cells and suggest that L1S may be useful to therapeutically manipulate immune responses.

## Results

### Lm expression of p60 enhances IFNγ production in cell cultures containing NK cells and infected DCs

**Figure 1:**
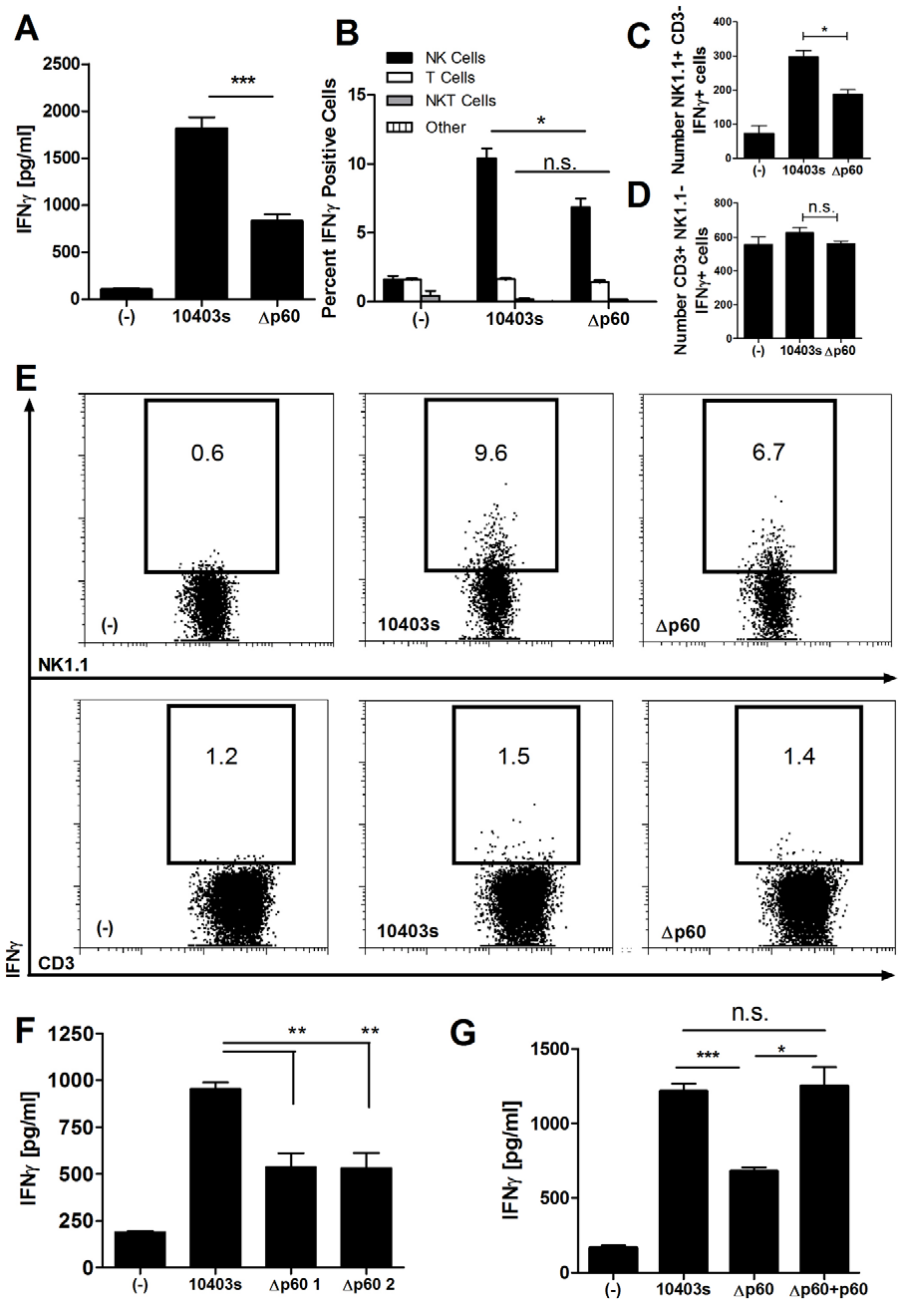
A secreted bacterial protein enhances activation of naïve mouse NK cells by *L. monocytogenes-*infected DCs. (A) BMDCs were infected in triplicate with wildtype Lm (10403s) or a mutant Δp60 Lm strain. NK-enriched NWNA splenocytes were added 2 h post-infection, and supernatants were harvested 21 h post-infection. The average ± SEM concentrations of IFNγ produced are plotted. (B-E) BMDC were infected with 10403s or Δp60 Lm, and NWNA cells were added 2 h post-infection. At 10 h post-infection, the NWNA cells were stained for CD3, NK1.1, and intracellular IFNγ. The average percentage (B) and Number (C,D) of IFNγ positive cells in gated NK1.1+CD3- (NK), NK1.1-CD3+ (T), CD3+NK1.1+ (NKT), or CD3-NK1.1- (other) cells are graphed ± SEM. (E) Representative dot plots are shown. (F) BMDC were infected in triplicate with wt Lm (10403s) or one of two independent Δp60 deletion mutants, and co-culture IFNγ was measured as in (A). (G) BMDCs were infected with wt or Δp60 Lm, or a Δp60 Lm strain complemented with His-tagged p60. Average ± SEM concentrations of IFNγ produced are shown. Data are representative of at least three (A-E,G) or two (F) experiments.

Systemic infections with Δp60 Lm strains elicit weak IFNγ production by NK cells [17]. Likewise, bone marrow dendritic cells (BMDCs) infected with Δp60 Lm elicited significantly less IFNγ from co-cultured naïve splenic lymphocytes (Figure 1A). Intracellular staining revealed that NK1.1+ cells were responsible for nearly all IFNγ production in these cultures (Figure 1B-E). Multiple independently generated p60 deletion mutants showed a similarly poor ability to induce IFNγ production in these co-cultures (Figure 1F). This weak IFNγ production was restored to wt levels when expression of p60 was restored in the Lm Δp60 mutant using an integrated vector coding for His tagged p60 protein (Figure 1G). The complemented Δp60+p60 strain secreted p60 at levels similar to wild-type Lm based on immunoblotting of culture supernatants (not shown). Reduced NK cell activation in response to Δp60 Lm infection might conceivably reflect reduced bacterial burdens within the infected BMDCs. However, microscopy and cfu plating revealed that the growth rate was identical for wt and Δp60 Lm, as was the percent of infected BMDCs over the course of infection (not shown). Finally, the ratio of cytosolic (actin-associated) versus phagosome localized Lm was also similar for the two strains (not shown). Thus, expression of p60 was not required for the invasion or cytosolic replication of Lm in BMDCs, but nonetheless increased the activation of neighboring NK cells.

### Purified p60 protein stimulates IFNγ production by NK cells in culture with primed DCs

**Figure 2:**
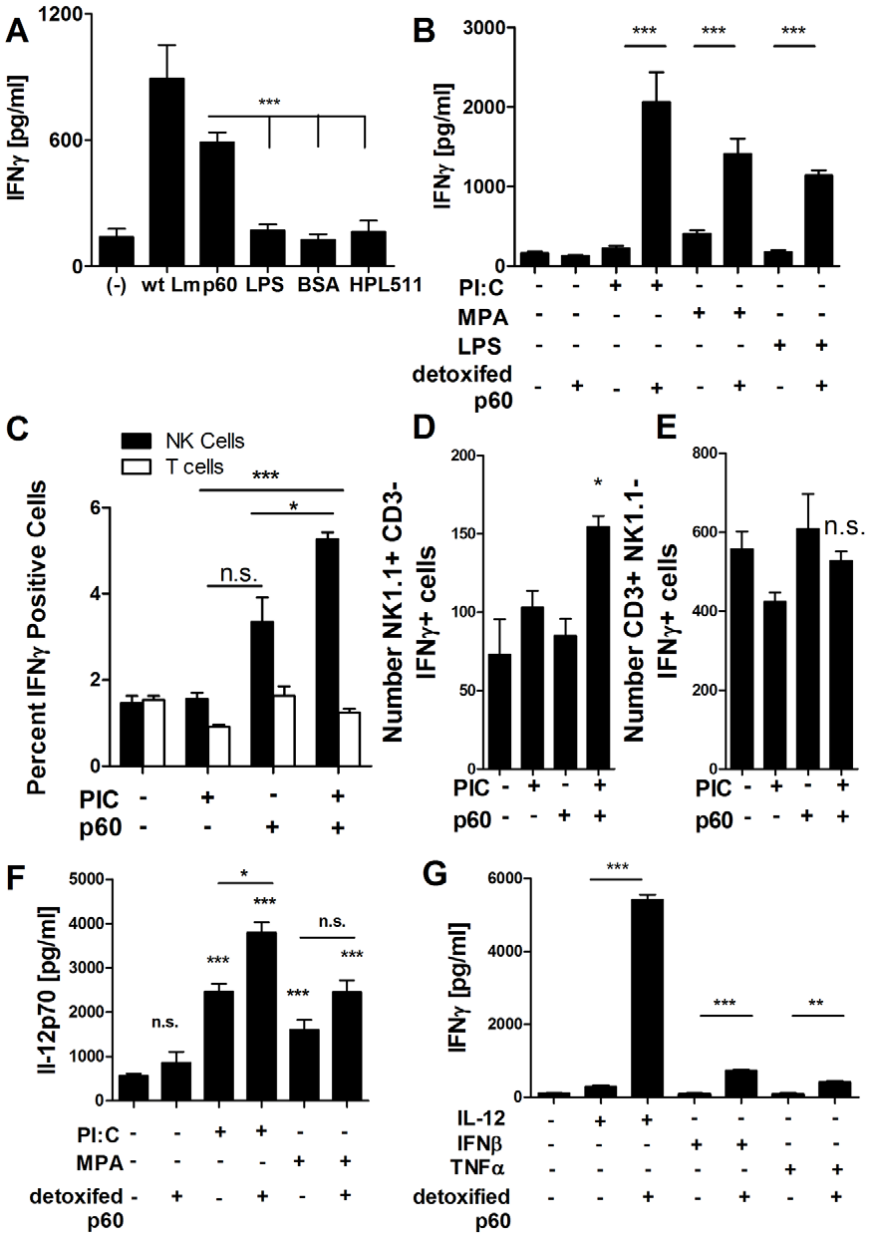
Primed DCs activate naïve NK cells when treated with purified p60 protein. (A) NWNA splenocytes were co-cultured in triplicate with BMDC infected with wt Lm or treated with 10 µg of recombinant His-tagged p60 purified from *E.coli*, 10 ng LPS, 10 µg BSA, or 10 µg of a His-tagged control protein, the phage autolysin HPL511. Average ± SEM concentrations of IFNγ produced are shown. (B) Purified p60 was detoxified of LPS using a polymyxin B column. BMDC in triplicate were primed for 3 h with 20 µg/ml poly I∶C, 10 ng/ml LPS, or 10 ng/ml MPA, and then treated with 10 µg of detoxified protein. NWNA splenocytes were co-cultured 2 h post-infection, and IFNγ was measured by ELISA 21 h post-infection/treatment. Average ± SEM concentrations of IFNγ produced are shown. (C-E) BMDC were pretreated 3 h with 20 µg/ml PIC, then stimulated with 10 µg detoxified p60. Two hours after p60 treatment, NWNA cells were added to the BMDC. Eight hours later, the NWNA cells were collected and stained for CD3, NK1.1, and IFNγ. NK cells are NK1.1 positive, CD3 negative, while T cells are CD positive, NK1.1 negative. The average percent (C) and number (D,E) of positive IFNγ cells are shown ± SEM. (F) Supernatants from BMDC and NWNA co-culture as in (B) were analyzed for IL-12 secretion by ELISA. Average ± SEM concentrations of IL-12 produced are shown. (G) BMDC were treated with 2 ng IL-12, 100 units IFNβ, or 2 ng TNFα with or without 10 µg detoxified p60. NWNA cells were added 2 h post-treatment, and IFNγ levels were measured by ELISA 21 h post-treatment. Average ± SEM concentrations of IFNγ produced are shown. (A-G) Data are representative of at least three experiments.

We expressed and purified recombinant His-tagged p60 protein from *E. coli* using nickel affinity and cation exchange columns. When added to co-cultures of BMDCs and nylon wool non-adherent cells (NWNA) prepared from naïve mouse spleens, the purified protein induced IFNγ production (Figure 2A). The recombinant p60 protein was associated with ∼1 ng of *E. coli* LPS per 1 µg of protein. However, this amount of LPS was insufficient to stimulate IFNγ production when added to the co-cultures without p60 protein (Figure 2A). Moreover, production of IFNγ was not seen in response to treatments with BSA or a His-tagged phage autolysin (HPL511) that was purified from *E. coli* using a similar procedure and also contained ∼1 ng LPS per µg protein (Figure 2A). To further exclude possible artifacts due to LPS, polymyxin B columns were used to remove LPS from the purified p60 protein. The detoxified p60 was initially insufficient to activate IFNγ production (Figure 2B), suggesting that activation by p60 required priming or maturation of the BMDCs. To test this, BMDCs were pretreated with TLR agonists for three hours before addition of p60. Pre-stimulation of co-cultures with LPS, the non-toxic LPS analog monophosphoryl LipidA (MPA), or poly I∶C (PIC) each sufficed to elicit IFNγ production following p60 stimulation (Figure 2B). None of the priming agents tested stimulated IFNγ production on their own.

Based on flow cytometry using intracellular IFNγ staining, NK cells were the major source of IFNγ produced in the co-cultures with primed and p60-stimulated BMDCs (Figure 2C-E). To test whether these NK cells responded directly to the stimulated BMDC, NWNA splenocytes were stained and flow sorted to obtain 97–98% pure populations of NK1.1+CD3- NK cells, CD3+NK1.1- T cells, and “other” cells (negative for both NK1.1 and CD3). Each sorted population was added to BMDCs (>90% CD11c+) that had previously been treated with LPS and a p60-derived peptide (peptide described further below). As previously shown for Lm-infected co-cultures [8], the purified NK cells produce IFNγ when cultured alone with stimulated BMDCs (Figure S1). The amount of IFNγ was not significantly affected by adding back either or both other cell populations present in NWNA splenocyte preparations (Figure S1). Although T cells did not impact IFNγ production by the NK cells, we observed small amounts of IFNγ production when purified splenic T cells were cultured alone with the stimulated BMDCs (Figure S1). This likely reflects the ability of memory CD8+ T cells to respond to IL-12 and IL-18 in the cultures [31] (see below for further discussion of cytokines present in the cultures). We conclude that the LPS and p60-stimulated BMDCs were sufficient to activate NK cells in these in co-cultures, and that the other cells present in the NWNA population did not significantly modulate this activation.

Stimulation of BMDC with LPS and other TLR stimuli elicits production of cytokines that stimulate DC and NK cells. Detoxified p60, failed to stimulate IFNγ production by the co-cultures in the absence of priming agents and also failed to induce significant levels of IL-12p70 secretion by BMDC. However, the priming agents PIC and MPA both elicited strong IL-12 production in the co-cultures containing NK cells and BMDC (Figure 2F). In some cases, but not universally, this IL-12p70 secretion was further enhanced by p60 stimulation. Recombinant IL-12p70, IFNβ, and TNFα each sufficed to prime the production IFNγ by detoxified p60 protein in the absence of TLR agonists (Figure 2G). IL-12 was by far the most potent priming agent, most likely due both to BMDC priming and the enhancement of IFNγ transcription in NK cells [32]. These findings suggested that cytokines produced in response to TLR agonists mediate priming or maturation of the BMDCs, which can then respond to recombinant p60 protein or mediate activation of naïve NK cells in NWNA splenocytes in response to this protein.

### Stimulation of IFNγ production from NWNA splenocytes by purified p60 protein requires co-culture with BMDCs and correlates with binding of the p60 protein to BMDCs

**Figure 3:**
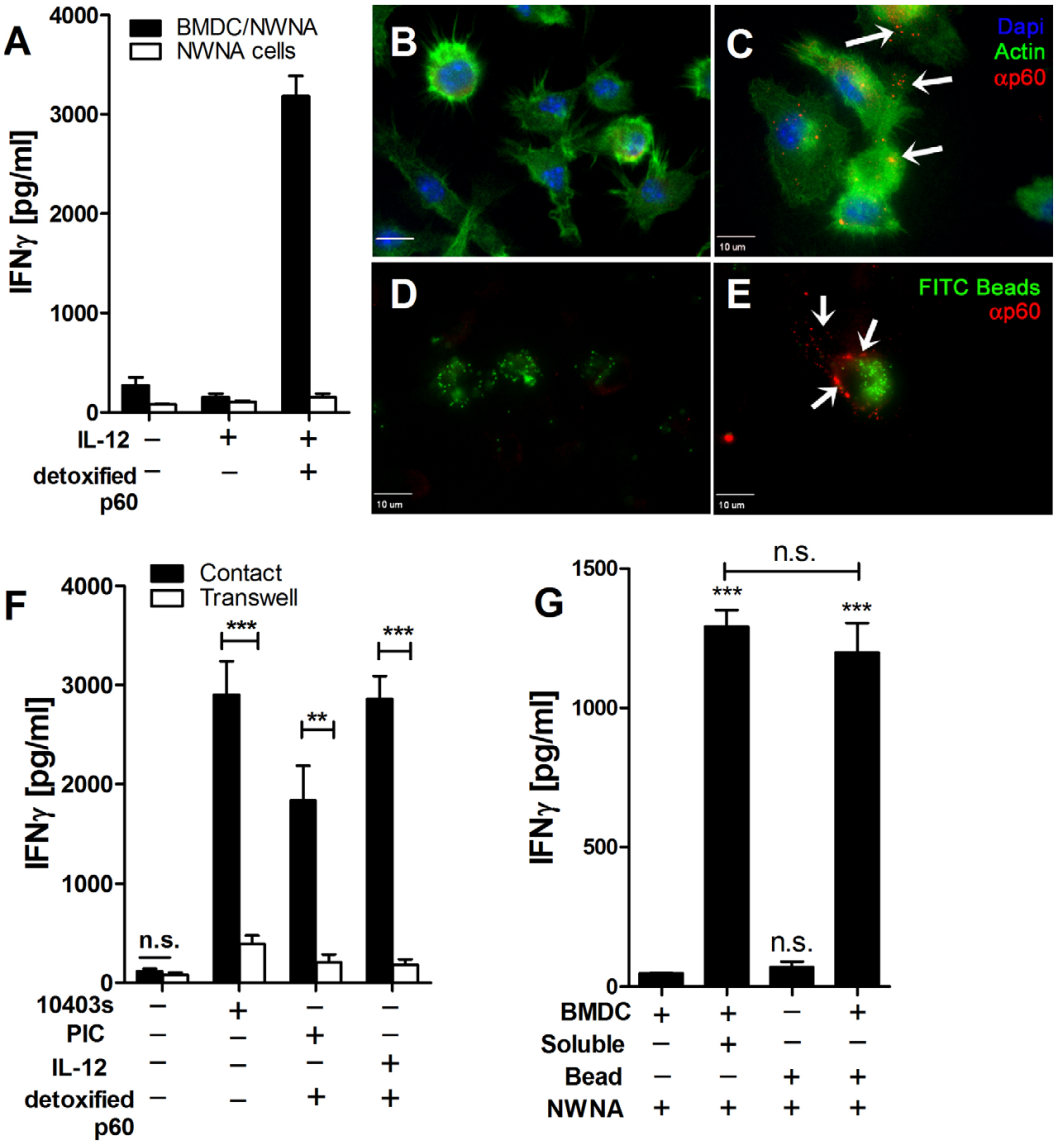
Purified p60 binds to BMDCs to active NK cells. (A) NWNA splenocytes were treated in triplicate with 2 ng IL-12 and 10 µg detoxified p60, in the presence or absence of BMDC. IFNγ in the supernatant was measured by ELISA 21 hours post-treatment. Average ± SEM concentrations of IFNγ produced are shown. (B-E) Scale bars represent 10 µm. BMDCs were untreated (B) or stimulated with 30 µg/ml p60 (C) for 4 h at 37°C and stained with polyclonal p60 antibody (red). Actin stained with Alexa-488 is shown in green, and nuclei (DAPI) are shown in blue. (D,E) BMDCs were treated with FITC-latex beads (green) alone (D) or with 30 µg/ml p60 protein (E) for 4 h at 37°C and stained with polyclonal p60 antibody (red). Arrows indicate p60 puncta. (F) BMDC were infected with WT Lm or primed with 20 µg/ml poly I∶C or 2 ng/ml IL-12 and treated with 10 µg detoxified p60. NWNA cells were added to the co-culture either in contact with the BMDC or separated by a 0.4 µm transwell support. Average ± SEM concentrations of IFNγ produced are shown. (G) NWNA splenocytes were cultured with BMDC alone or treated with 10 ng soluble LPS + 10 µg soluble p60, or with Ni beads and an equal amount of p60 and LPS (Beads). IFNγ in the supernatant was measured by ELISA 21 hours post-treatment. Data shown are representative of two (G) or at least three experiments (A-F).

We next asked how p60 might mediate NK cell activation in co-culture by examining the role of accessory DCs. Addition of p60 protein did not stimulate IFNγ production in the absence of NK cells or when added to NWNA cells in the absence of BMDCs ([17] and Figure 3A). This result suggested two possibilities. Either p60 protein might act on DCs to induce the ability of DCs to activate NK cells, or the protein might be presented to NK cells by DCs for NK cell activation. To investigate whether p60 protein bound to BMDCs, the cells were treated or not with p60 protein, fluorescent beads, or p60 plus beads. After washing, the treated and untreated BMDCs were stained using anti-p60 rabbit polyclonal antisera and a secondary Cy3-labeled anti-rabbit Fab (Figure 3B-E). A punctuate staining pattern was seen on the stained p60-treated BMDCs (Figure 3C and E). Identical results were obtained using two independent anti-p60 polyclonal antibodies (data not shown). This punctuate staining was not observed on stained untreated cells or cells treated with beads alone (Figure 3B and D), nor on sorted NK cells, T cells, or other NWNA splenocytes (not shown). The punctuate staining for p60 did not require detergent permeation of the BMDC membrane (not shown), nor did p60 puncta co-localize with phagocytosed FITC-labeled latex beads (Figure 3E). These data suggest that p60 protein binds to an unknown receptor/s present at or near the surface of BMDCs.

We previously reported that contact between DCs and NK cells was required for NK cell activation during Lm-infection [8]. Similarly, contact between the DC and NWNA splenoctyes was required for p60-induced NK cell activation (Figure 3F). It was conceivable that binding of p60 to the DC surface might permit presentation of this protein to NK cells. However, nickel beads coated with a His-tagged p60 were not able to stimulate NWNA cells in the absence of BMDC (Figure 3G). Together, these data suggested that p60 primarily stimulates NK cell activation indirectly, due to its effect on DCs.

NK cells might respond to altered MHC I expression and/or upregulation of stress ligands by BMDCs treated with p60 protein [5], [33]. Thus, we stained BMDCs that had been primed with LPS plus or minus an active p60-derived peptide (described further below) and assessed their expression of activation markers (MHCII) and several known ligands for NK cell surface receptors (Figure S2). MHCII expression increased after protein treatment, consistent activation of the BMDC. No down regulation of MHC I was observed and the expression of NKG2D ligands RAE1γ, RAE1δ, and MULT1 were unchanged. There was no change in staining levels for the SLAM family members 1, 2, 3, and 6. SLAMF 5 staining was slightly reduced after protein treatment, which is likely due to DC activation. These data suggested that NK cell activation by p60 was due to effects of p60 on DCs that were independent of altering expression of these known ligands for NK cell activating and inhibitory receptors.

### Treatment with p60 causes BMDCs to secrete IL-18, which is required for IFNγ production by co-cultures containing NK cells

**Figure 4:**
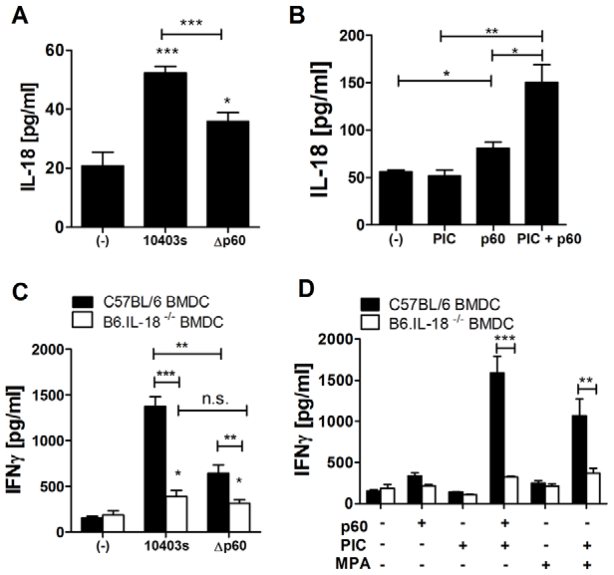
IL-18 produced by BMDCs is required for p60-elicited IFNγ from NK cells in co-culture. All treatments were performed in triplicate; average cytokine concentrations ± SEM are shown. (A) BMDCs were infected with 10403s or Δp60 Lm, and supernatant levels of IL-18 were assessed by ELISA 21 hours post-infection. (B) BMDC were treated with 20 µg/ml PIC and 10 µg detoxified p60 alone or in combination, and supernatant levels of IL-18 were assessed by ELISA 21 hours post-infection. (C,D) BMDC from C57B6 or *IL-18^-/-^* mice were infected with 10403s or Δp60 Lm (C) or treated with 10 ng/ml MPA or 20 µg/ml PIC alone or in combination with 10 µg detoxified p60 (D). Two hours post treatment, C57B6 NK-enriched NWNA splenocytes were co-cultured with the BMDCs. IFNγ levels were measured by ELISA 21 hours post-infection. Data shown are representative of at least three experiments.

Both cell contact and inflammatory cytokines such as IL-12 and IL-18 modulate NK cell activation and IFNγ production [34]. IL-12 production by BMDC infected with wildtype versus Δp60 Lm was not significantly different (data not shown). Since IL-18 production is essential for NK cell activation by Lm infected BMDCs [8], we asked whether bacterial expression of p60 effected IL-18 production in infected BMDCs. We found that secretion of IL-18 was significantly reduced in the supernatants of C57BL/6 BMDCs infected with Δp60 Lm (Figure 4A). Consistent with this observation, detoxified p60 protein in combination with PIC strongly simulated IL-18 secretion from BMDCs (Figure 4B). We next evaluated the effects of IL-18 production on IFNγ production in cultures of infected BMDC and NWNA splenocytes. In response to Lm infection, *IL-18^-/-^* BMDCs stimulated very little IFNγ production (Figure 4C). Moreover, the amount of residual IFNγ produced in these co-cultures was no longer affected by bacterial expression of p60. Further, IL-18 expression in BMDCs was additionally required to elicit IFNγ production in co-cultures primed with PIC or MPA and stimulated with detoxified p60 protein (Figure 4D). Together, these data suggest that binding of p60 to BMDC elicits IL-18 secretion, which is required for activation of NWNA splenocytes.

### The enzymatic activity of p60 is not required for its ability to stimulate IFNγ production in co-cultures of BMDCs and NWNA cells

The p60 protein has been shown to weakly digest peptidoglycan (PGN) [21], [29], hence, we previously hypothesized that PGN cleavage by p60 might release muramyl di-peptide (MDP) or other bioactive muropeptides [21], [29]. MDP is detected by NOD2, which signals through the RIP2 kinase [35], [36], [37], [38]. To test whether MDP generation by p60 might stimulate NK cell activation, we compared the ability of Lm infected B6 and *B6.RIP2^-^* ^/-^ BMDC to activate NK cells from B6 mice. Bacterial expression of p60 enhanced IFNγ production in NWNA splenocytes co-cultured with *RIP2^-/-^* BMDCs to the same extent as C57B6 BMDCs (Figure S3A). Additionally, purified recombinant p60 stimulated BMDC and NK cell enriched splenocytes co-cultures in the absence of added *Listeria* PGN. Therefore, generation and detection of the MDP PGN fragment was not required for NK cell activation nor for the ability of p60 to enhance such activation.

**Figure 5:**
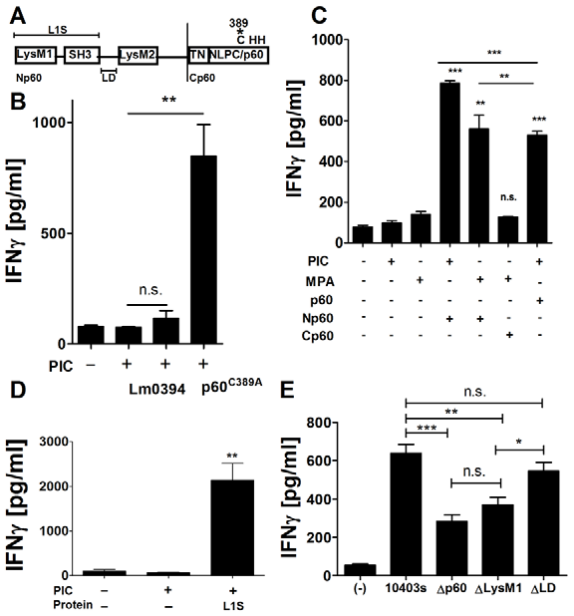
The p60 L1S fragment is sufficient to activate DC/NK cell co-cultures. (A) p60 domain map. The p60 protein consists of two LysM domains on either side of an SH3 domain in the N terminal portion. The C-terminus consists of an NLPC/p60 domain preceded by a TN repeat region. C389, H439 and H465 are the predicted catalytic triad. Recombinant versions of (B) p60^C389A^, the Lm p60 homolog 0394, and (C) the p60 fragments Np60, Cp60, and (D) LysM1-SH3 (L1S) were purified and detoxified of LPS using a polymyxin B column. BMDC were primed for 3 h with 20 µg/ml poly I∶C or (C,D) 10 ng/ml MPA (C) then treated with 10 µg of detoxified protein. NWNA splenocytes were co-cultured 2 h post-treatment, and IFNγ was measured by ELISA 21 h post-treatment. (E) BMDC were infected with wt 10403s, Δp60, ΔLysM1, or ΔLD Lm. NWNA splenocytes were added at 2 hr post-treatment. IFNγ levels were assessed 21 hr post-treatment by ELISA. Data are representative of at least three (B-D) or two (E) experiments. All treatments were performed in triplicate.

Like the *Bacillius subtilis* LytF protein, p60 contains a C-terminal NLPC/p60 domain with a putative catalytic triad of two histidines and a single cysteine residue (Figure 5A). In LytF, the cysteine is essential for endopeptidase activity and permits cleavage of the cross-linking peptide chains in peptidoglycan (PGN) [24]. However, NLPC/p60 domains have also been associated with other catalytic functions. To formally test whether the enzymatic activity of p60 was required for stimulation of NK cell activation, we engineered and purified a p60 derivative in which the catalytic cysteine residue was mutated to alanine. The resulting p60^C389A^ mutant protein was purified as for wt p60 and tested for digestion of heat-killed Lm and crude Lm PGN substrates using zymography (Figure S3B and not shown). As previously published [21], [29], the wt p60 protein cleaved PGN, although this activity was much weaker than that seen with a control phage lysin (HPL511). The p60^C389A^ was completely inactive in this assay (Figure S3B), confirming that the cysteine residue was required for PGN digestion by p60. Nonetheless, the purified p60^C389A^ was as efficient as the catalytically active wt protein for stimulating IFNγ production in co-cultures of NWNA splenocytes and BMDCs (Figure S3C, Figure 5B). These data demonstrate that the enzymatic activity of p60 is not required for its ability to promote NK cell activation.

### The N-terminal LysM-SH3 region of p60 is sufficient to stimulate IFNγ production by NWNA cells

Given that enzymatic activity was dispensable for NWNA splenocyte activation by p60, we asked whether this activation was associated with NLPC/p60 or other domains. The Lm genome contains a homolog of p60 (Lm0394) with both an SH3 domain and a C-terminal NLPC/p60 domain but lacking the N-terminal LysM domains found in p60. A His-tagged recombinant Lm0394 protein was unable to activate NWNA splenocytes in co-culture (Figure 5B). Thus, the presence of SH3 and NLPC/p60 domains was not sufficient to confer the ability to activate co-cultures. Additional p60 derivatives were engineered and purified, including an N-terminal fragment (Np60) truncated immediately before the TN repeat region and a C-terminal fragment (Cp60) that comprised the TN repeats and NLPC/p60 domain (Figure 5A). These truncated proteins were purified, detoxified, and tested as for full length p60. Np60 induced IFNγ production in co-cultures pre-stimulated with either PIC or MPA, while Cp60 failed to induce IFNγ (Figure 5C). Further truncation of the N-terminal region mapped the stimulating activity to a fragment containing the LysM1 and SH3 domains, termed L1S (Figure 5D). The results of our experiments with SH3-domain-containing Lm0394 indicate that the LysM1 domain may be responsible for the activity of L1S. However, efforts to purify the LysM1 or SH3 domains alone have thus far been unsuccessful, suggesting that both domains may be required for conformation and stability. Given that the L1S polypeptide was the minimal active component of p60 identified in our studies, we tested whether the LysM1 domain was necessary for p60-induced co-culture activation during Lm infection. We compared Δp60 mutants complemented with p60 constructs that lacked the LysM1 domain or the linker domain (LD) between the SH3 and LysM2 domains (Figure 5A). Both complemented strains expressed and secreted the p60 mutant proteins at levels comparable to wildtype Lm based on immunoblotting of precipitated culture supernatants (not shown). The ΔLysM1 complementation mutant induced low IFNγ levels in co-culture similar to Δp60 Lm infection, while the ΔLD complementation mutant induced IFNγ similar to wild type Lm infection (Figure 5E). Thus, the LysM1 domain appears to be largely responsible for p60-mediated activation of BMDC/NWNA splenocyte co-cultures.

### L1S activates NK cells *in vivo*

**Figure 6:**
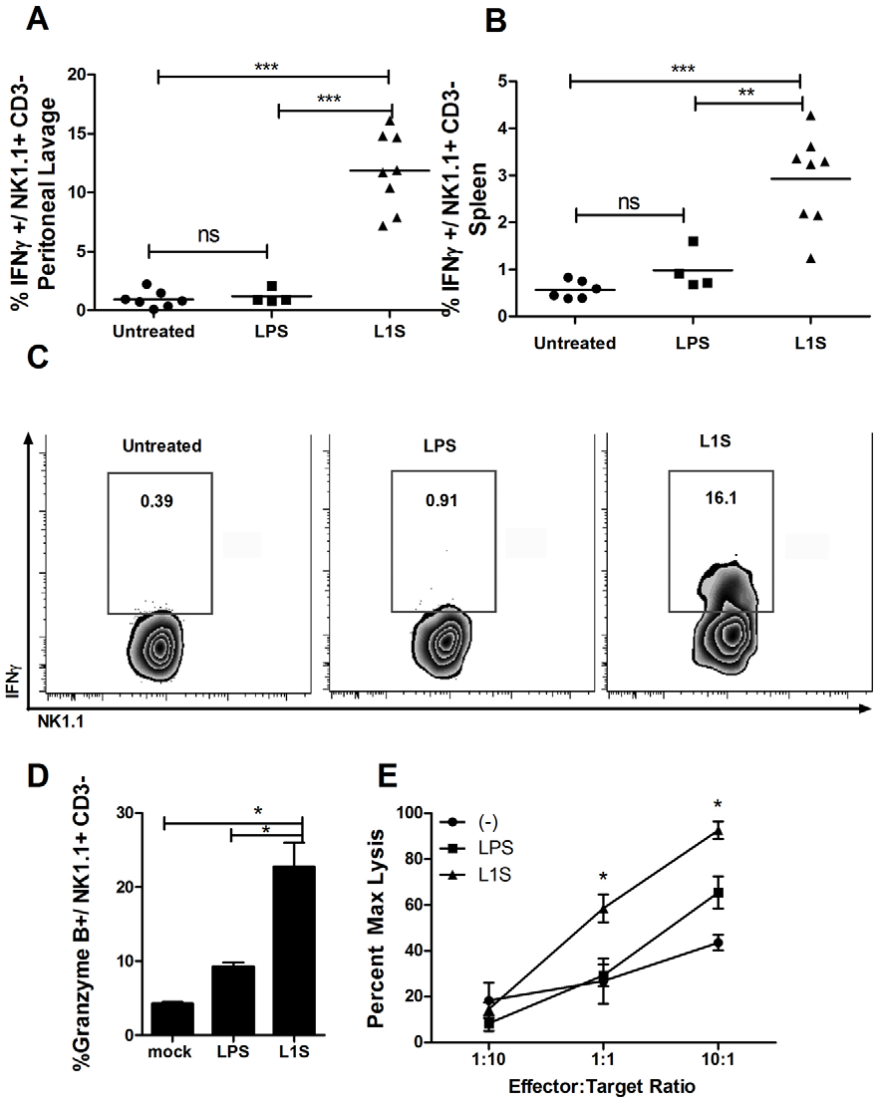
L1S activates NK cells *in vivo* to secrete IFNγ and increase cytotoxicity. Mice were injected i.p. with 500 µg L1S or 500 ng LPS in 250 µl PBS. After 24 hours (A) peritoneal cells harvested by lavage and (B) splenocytes were stained for CD3, NK1.1, and intracellular IFNγ. Shown are graphical representations of the NK1.1+, CD3- cells that stained positive for IFNγ. Symbols represent individual mice. (C) FACS plots showing the IFNγ positive gate used for (A and B). Gated NK cells from peritoneal lavage are depicted. Data are pooled from two independent experiments; n =  two to four treated mice per experimental group. (D) The peritoneal cells from (A) were stained for granzyme B. The average percent granzyme B-positive NK1.1+/CD3- cells are shown. (E) NK-enriched splenocytes were co-cultured with BMDC that were treated with LPS with or without L1S for 21 hours. The NWNA splenocytes were added to B16F10 melanoma target cells at the effector∶target ratios indicated, based on estimated 5% NK cells in the splenocytes. Cytotoxicity was assessed after 4 hours incubation. Conditions were assessed in triplicate, and results are representative of two experiments.

The regulation of NK cell activation and responses *in vivo* may differ from their regulation in our cell culture system. We thus asked whether purified, LPS-associated L1S was sufficient to activate NK cells *in vivo* when administered to mice by intraperitoneal (i.p.) injection. LPS was administered to a second group of mice as a negative control. At 24 h after injecting the L1S or LPS, IFNγ production by both splenic and peritoneal infiltrating NK cells was assessed using intracellular cytokine staining. The data showed that LPS treatment failed to stimulate NK cell activation in the absence of L1S polypeptide. However, there were significant increases in the percentage of NK1.1^+^CD3^-^ cells staining positive for IFNγ in both peritoneum (Figure 6A, 6C) and spleen (Figure 6B). The activation of splenic NK cells was more modest than seen in the peritoneum, suggesting the NK cell activation largely occurred locally at the site of L1S injection (Figure 6B). The NK cell activation by LPS-associated L1S was dose-dependent (Figure S4). We additionally observed that the percent granzyme B positive NK1.1+CD3- NK cells was increased in the peritoneal cells in response to L1S treatment (Figure 6D). Hence, we measured cytotoxicity from NWNA splenocytes after co-culture with BMDCs stimulated with LPS with or without L1S. Consistent with the increased granzyme B staining *in* vivo, L1S significantly enhanced the cytolytic activity of NWNA splenocytes against NK cell-sensitive B16F10 melanoma target cells *in vitro* (Figure 6E). These data confirmed that the p60-derived polypeptide was bioactive in the treated animals and suggested that L1S might be useful for therapeutic stimulation of both cytokine and cytoxicity-based immune responses.

### *In vivo* administration of L1S confers protection against *Francisella* infection

**Figure 7:**
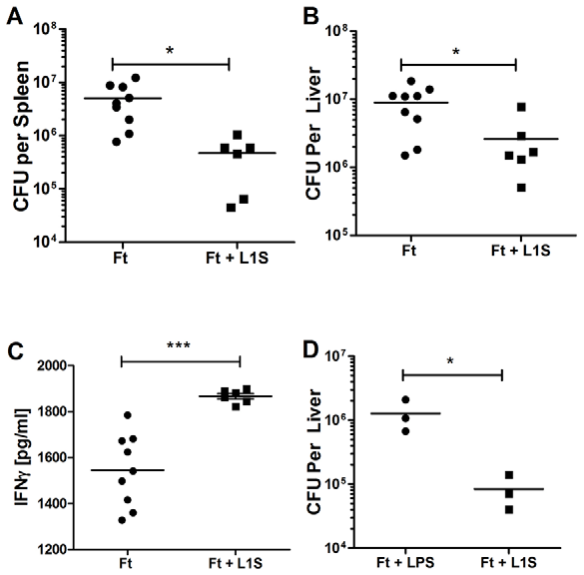
*In vivo* administration of L1S is protective for *Francisella* infection. (A-C) Mice were pretreated with 300 µl PBS control or 500 µg purified L1S injected i.p. After 24 hours, the mice were infected i.p with 10^4^ live *F. tularensis* LVS. CFUs were counted from the spleens (A) and livers (B) of the infected mice 4 days post-infection. (C) IFNγ from serum was measured by ELISA. Data are pooled from two independent experiments; n =  three to six mice per experimental group. (D) Mice were pretreated with 500 ng LPS with or without 500 µg L1S in 300 µl PBS injected i.p. 24 hours before infection with 10^4^ live *F. tularensis* LVS, administered i.p. CFUs from liver were assessed 4 days post-infection.

Secretion of IFNγ by NK cells is thought to promote clearance of the bacterial pathogen *Francisella tularensis* [39], [40], [41]. However, this cytosolic intracellular bacterial pathogen normally suppresses innate immune responses [41], [42], [43]. We thus hypothesized that boosting of NK cell activation during *F. tularensis* infection might reduce host susceptibility to this pathogen. To test this hypothesis, we administered purified, LPS-associated L1S or PBS alone by a single i.p. injection 24 hours prior to an i.p. infection with the attenuated live vaccine strain of *Francisella tularensis holarctica* LVS (Ft). Bacterial burdens in the infected spleens (Figure 7A) and livers (Figure 7B) were assessed 96 hours post Ft infection. Colony-forming units (CFU) recovered from spleens and livers of the L1S treated mice were significantly reduced when compared to the control mice. Consistent with the increase in IFNγ+ NK1.1+CD3- cells seen after *in vivo* L1S stimulation (Figure 6A, 6B), we observed a significant increase in serum IFNγ levels in the mice treated with L1S prior to Ft infection (Figure 7C). To control for the potential effects of LPS associated with purified L1S, we pre-treated mice with LPS or LPS-associated L1S 24 hours prior to Ft LVS infection as above. The CFUs recovered 4 days post-infection were significantly lower in mice pre-treated with LPS-associated L1S compared to LPS alone (Figure 7D). Serum levels of IFNγ were also significantly higher in the L1S versus LPS pre-treated mice (not shown), which correlates with the observed minimal effect of LPS on IFNγ levels in NK cells *in vivo* (Figure 6A, 6B). These findings suggest that p60 and its derivatives enhance NK cell activation in a biologically relevant manner and may be useful for further development as a therapeutic for immune stimulation.

## Discussion

Bacterial pathogens have developed numerous strategies to interfere with or subvert host immune responses [44], [45]. Our findings here demonstrated an indirect role for the abundantly secreted *L. monocytogenes* (Lm) p60 protein in modulation of NK cell activity. We showed that Lm secretion of the p60 protein during infection of cultured BMDCs stimulated enhanced activation of naïve NK cells in cell co-culture assays. Moreover, endotoxin-free purified p60 protein was sufficient to stimulate IFNγ production from NK cells in co-cultures containing BMDCs primed with TLR agonists or inflammatory cytokines such as IL-12. Purified p60 protein bound to the BMDCs and in the presence of priming stimuli this binding correlated with BMDC secretion of the NK cell activating cytokine IL-18. These findings support the model that p60 indirectly activates NK cells by stimulating a DC surface receptor in a manner that induces secretion of IL-18. Consistent with this model, IL-18 production by the BMDCs was essential for eliciting IFNγ production by NK cells and cultures of NWNA splenocytes. The known endopeptidase enzymatic activity of p60 was not required for this biological response and stimulation of NK cell activation by p60 or its derivatives was independent of bacterial PGN and muropeptide detection systems dependent on the RIP2 kinase. Rather, the ability to stimulate DC-dependent NK cell responses mapped to an N-terminal fragment of p60 that contains a LysM domain and a bacterial SH3 domain. A polypeptide containing just these domains (L1S) was sufficient to stimulate DC-dependent NK cell activation both in cell culture assays and when administered to mice in the absence of Lm infection. These results thus revealed a novel role for a bacterial LysM domain-containing protein in the modulation of mammalian innate immune responses.

Our studies here demonstrated that extracellular delivery of p60 protein or the L1S polypeptide in cell culture acted in concert with DCs to stimulate NK cell activation. However, the p60 protein was not sufficient to activate NK cells in the absence of primed BMDCs. In addition, soluble L1S polypeptide triggered NK cell activation when injected into mice without any known mechanism for uptake into the cytosol of host cells. These data suggest that p60 and/or L1S act extracellularly to increase the ability of DCs to promote NK cell activation. Consistent with this interpretation, we observed that p60 protein bound to the surface of BMDCs but not NK cells. Staining of p60 on BMDCs that were fed latex beads suggested that aggregates of p60 are not simply phagocytosed. Furthermore, delivery of p60 into the cytosol of cultured BMDCs using transfection protocols did not improve NK cell activation (not shown). These data suggest that p60 protein acts extracellularly to promote NK cell activation. The fact that infected individuals develop antibodies against p60 further suggests this protein may be abundant extracellularly during Lm infection [31]. Potential sources of extracellular p60 include production by extracellular bacteria, which are known to be present at early and later times of infection [46], or release of protein upon lysis of infected cells. However, we cannot exclude the possibility that p60 present in the cytosol after phagosomal escape of Lm also contributes to NK cell activation. Indeed, p60 protein is abundant in the cytosol of Lm infected macrophages and stimulates protective cytotoxic T cell (CTL) responses [47], [48]. Since cytokines and TLR agonists are also present during Lm infections, soluble extracellular p60 protein that interacts with DCs or other infected cells during *in vivo* Lm infection is likely an important stimulus for NK cell activation during *in vivo* Lm infection. However, our data here (Figure 1) and in a prior publication [17] clearly indicate that there are also p60-independent mechanisms for NK cell activation.

The activation of naïve NK cells by DCs infected with live Lm bacteria was previously shown by us and others to require both direct contact between DCs and NK cells and the production of IL-12 and IL-18 [8], [18]. Lm bacteria obviously contain TLR agonists that can induce IL-12 production to prime NK cell activation during *in vivo* infection. However, it has not been clear whether specific bacterial factors stimulate IL-18 production and/or cell contact between naïve NK cells and DCs. Our data here implicate the L1S region of p60 as a bacterial factor that promotes IL-18 production by DCs. Specifically, we showed that priming of BMDCs with TLR agonists stimulated IL-12p70 production by these cells and that IL-12p70 could substitute for TLR agonists. In some experiments, we also observed a modest p60-induced enhancement of IL-12 secretion from BMDCs that were already primed with TLR agonists, which is consistent with the ability of IL-18 to positively regulate IL-12 production. However, neither TLR agonists nor IL-12 were sufficient to stimulate NK activation in the absence of p60 protein and the IL-18 production elicited by p60. Moreover, despite the presence of IL-12 and IL-18, stimulation of BMDCs with TLR agonists and p60 was insufficient to stimulate NK cell activation when there was not direct cell-cell contact between the BMDCs and the NK cells. The p60 treatment appeared to induce a more activated phenotype in BMDCs but it did not alter the expression by BMDCs of several known ligands for NK cell activating and inhibitory receptors. Thus, there exist at least three possible explanations for the contact requirement: (1) The p60 stimulation triggers both IL-18 secretion and expression of an activating ligand by the BMDCs. This ligand is not one we have tested and may be novel. (2) Contact merely serves to increase the local concentration of IL-18 (and perhaps IL-12) above some threshold that normally prevents activation of the naïve NK cells. This may be facilitated by immunological synapses formed between the DC and NK cells, as previously suggested [13], [14]. (3) BMDCs constitutively express (or are induced to express e.g. by p60 or IL-12) a surface associated “co-stimulatory” factor that is required to “prime” the NK cells for responsiveness to IL-18. Ongoing and future studies focused on identification of putative ligands or co-stimulatory factors may resolve which, if any, of these possible explanations is correct.

NK cells are the major source of IFNγ production early after viral and bacterial infections. IFNγ normally plays a protective role in immunity to Lm and other pathogens, including *F. tularensis* (Ft). IFNγ induces CD4 Th1 differentiation, stimulates cytotoxic CD8 cells, and activates macrophages to become more bactericidal [49], [50]. During Ft infection, IFNγ-positive NK cells are quickly recruited to sites of infection, where they promote granuloma formation and limit bacterial spread [39], [41]. We found that injection of L1S polypeptide into mice was sufficient to activate NK cells to produce IFNγ, particularly at the site of injection. We also found increased serum levels of IFNγ persisting through infection in mice pre-treated with L1S polypeptide. Presumably, the IFNγ produced by these NK cells created a non-permissive environment for Ft expansion. Thus, when Ft was inoculated at the same site as the L1S polypeptide, its growth was significantly reduced compared to inoculations in the absence of L1S. It will be important to determine whether L1S polypeptide injection might also protect against other routes of Ft infection and against other pathogens.

In contrast to Ft infection, the results of *in vivo* depletion studies suggest that NK cells are associated with increased susceptibility of mice to Lm [17], [51], [52], [53], [54] and the expression of p60 by Lm increased host susceptibility to systemic Lm infection [17], [21]. Thus, production by Lm of a protein that promotes NK cell activation correlates with the fact that NK cell activation increases susceptibility to Lm. It was also previously reported that IFNγ production by NK cells fails to protect mice against systemic Lm infections [55]. This may be due to suppression of macrophage responsiveness to IFNγ during early stages of Lm infection [56]. Thus, Lm produces a protein that enhances NK cell activation and also has been shown to be more pathogenic in the presence of NK cells. It will thus be of interest in future studies to understand the mechanisms by which activated NK cells promote Lm pathogenicity.

In contrast to Lm, Ft normally suppresses host inflammatory responses during the initial stages of infection [42], [43]. The Ft genome contains several LysM-containing proteins, but using BLAST searches we failed to identify any Ft proteins whose LysM-domains showed more than 20% identify to the LysM1 region of p60. Thus, it is possible that the LysM proteins present in Ft have evolved to lack residues critical for binding to DCs or activation of IL-18 secretion by DCs. Consistent with this model, we found that no IFNγ was produced by NWNA splenocytes cultured with Ft-infected BMDCs (data not shown). However, this issue will need to be further investigated, since it is also possible that Ft LysM proteins are not secreted and thus accessible to bind DCs in the same manner as the Lm p60 L1S region.

NK cells are attractive targets for therapy in cancer and infectious diseases as they can directly kill target cells. NK cells also regulate immune and autoimmune B cell and T cell responses through production of IFNγ or inhibitory cytokines such as TGFβ and IL-10 [57], [58]. NK cells have additionally been shown to impact Type I diabetes, multiple sclerosis, and other diseases associated with inflammation [59], [60]. Our findings demonstrated use of the p60 protein to stimulate activation of cultured NK cells. L1S also demonstrated effective NK cell activation when administered *in vivo*. With refinement, p60 or L1S may be adapted to therapeutic use to harness anti-cancer or immune regulatory effector mechanisms of NK cells. Further experimentation on the clinical and biological effects of p60 protein may thus provide novel approaches to manipulate host immune responses. Additionally, it will be of interest to determine whether and how LysM-containing proteins from other pathogens modulate innate immune responses. Such studies should improve our understanding of bacterial pathogenesis and the role of NK cells in immune responses.

## Methods

### Ethics statement

This study was carried out in strict accordance with the recommendations of the Public Health Service Policy on the Humane Care and Use of Laboratory Animals, the Guide for the Care and Use of Laboratory Animals, and the Association for Assessment and Accreditation of Laboratory Animal Care. The protocols used were approved by the Institutional Animal Care and Use Committee at National Jewish Health (Protocol Permit AS2682-9-13). All efforts were made to minimize suffering.

### Mice

C57BL/6 and B6.*IL-18-/-* mice were obtained from Jackson labs. Breeders of B6.*Rip2^-/-^* mice were generously provided by K. Kobayashi (Dana-Farber/Harvard, Boston, MA). Mice were bred and housed in the Biological Research Center of National Jewish Health. Studies were performed with the approval of the National Jewish Health Institutional Animal Care and Use Committee.

### Bacterial strains

Wild type *Listeria monocytogenes* 10403s was used in these studies. In-frame deletion of p60 in 10403s was done by allelic exchange, as described [21]. The full p60 complementation mutant expresses a secreted His-tagged p60 protein expressed from the pPL2-derived vector pIMK2, a generous gift from C.G.M. Gahan described in [61]. The ΔLysM1-p60 complementation mutant lacks the first LysM1 domain, residues 26-69, and is also expressed from the pIMK2 vector. SOE PCR primer sequences are provided in Table S1. 10403s Δp60 was transformed with the His-p60 construct or ΔLysM1and p60 protein secretion was assayed by immunoblot of TCA precipitated of supernatants from overnight Lm cultures. Plasmid DNA encoding the ΔLD-p60, lacking residues 138-179, was provided by E. Pamer (Sloan-Kettering, NY) and described in [62]. The mutated gene was amplified with primers described in Table S1 and subcloned into the pPL-2 vector for transformation into 10403s Δp60 Lm. The *Francisella tularensis* live vaccine strain (LVS) holarctica type b was obtained from ATCC BEI Resources (Manassas, VA). *Escherichia coli* TOP10 cells were obtained from Invitrogen (Carlsbad, CA) and were used to clone and express all His-tagged purified proteins in this study.

### BMDC culture and infection

Femoral bone marrow was flushed and cultured in RPMI 1640 (high glucose) (Gibco, Invitrogen) with 10% FBS, .1% betamercapto-ethanol, 1%L-glutamine, 1% sodium pyruvate, 1% penicillin/streptomycin, and 2 ng/ml GM-CSF. BMDC were washed on days 2 and 4, and harvested on day 7. 3×10^5^ cells were plated per well of a 24-well plate in triplicate for >12 hours in antibiotic-free media, then infected with log phase 10403s wt or Δp60 at MOI of 1 for 1 hour. Cells were then washed and treated with 10 µg/ml gentamycin. For protein stimulation, 3×10^5^ BMDC were treated with 10 µg purified protein plus or minus pre-treatment with 10 ng/ml ultra-pure LPS, 10 ng/ml mono-phosporo-Lipid A (MPA) (Sigma-Alderich, St. Louis, MO), or 20 µg/ml Polyinosine-polycytidylic acid (PIC) (Invivogen, San Diego, CA) for 3 hours.

### Co-Cultures for NK cell activation

Splenocytes were prepared and enriched for lymphoctyes by nylon wool non-adherence (NWNA) as described [8]. Lymphocytes were 5-6% CD3^-^ NK cells based on staining with NK1.1 (PK136) and CD3 (145-2C11) (BD Biosciences Franklin Lakes, NJ and eBioscience San Diego, CA). The splenocytes were added to the BMDC at a 0.1∶1 NK cell∶BMDC ratio at 2 hours post-infection. To obtain purified NK and T cells from NWNA splenocytes, cells were stained with NK1.1 and CD3 and sorted by flow cytometry on the Synergy (Icyt, Champaign, IL). Purified NK1.1+/CD3- NK cells (3×10^4^), CD3+/NK1.1- T cells (5×10^4^) and NK1.1-/CD3- cells (3×10^4^) were added to 3×10^5^ BMDC per well. To test NWNA splenocytes activation in the absence of BMDC, a 50% bead slurry of Ni-NTA agarose beads (Invitrogen) was washed 5 times with PBS, associated with 50 µg L1S/well, washed 2 times with PBS, and then was added to NWNA splenocytes in the presence or absence of BMDC.

### Protein purification

DNA coding for the mature p60, p60^C389A^ , Lm 0394, Np60, Cp60, and L1S were cloned into the pTrcHis-TOPO TA cloning vector (Invitrogen, Carlsbad, CA) for IPTG-induced expression in TOP 10 *E.coli*. Primers are listed in Table S1. The phage autolysin HPL511 was purified from a construct supplied by M. Loessner (Zurich). *E.coli* were lysed with BugBuster (Novagen, Gibbstown, NJ) in 20 mM Na phosphate, 0.5 M NaCl, and 20 mM imidazole, pH 7.4, containing protease inhibitor and 2 mg/ml lysozyme. Proteins were purified using HisTrap FF 5 ml affinity columns (GE, Piscataway, NJ) on an Akta FPLC (GE). Further purification was achieved with Hi-Trap FF or HP (GE) cationic exchange in 50 mM HEPES buffer. LPS was removed from the proteins using polymyxin B columns as indicated by the manufacturer (Thermo Scientific, Waltham, MA).

### ELISA

Supernatant levels of murine IFNγ, IL-12p70, and IL-18 were measured at 21 hours post-infection using commercial ELISA kits (BD Biosciences, MBL International, Woburn, MA).

### Microscopy

BMDCs (3×10^5^ per coverslip) were treated with 30 µg/ml purified p60 with or without 1×10^8^ FITC-labeled 0.5 um latex beads (Polysciences, Inc, Warrington, PA). p60 was probed with PFII rabbit anti-p60 (supplied by E. Pamer, New York) and Fab (ab′)2 goat-anti-rabbit Cy3 (Invitrogen). Actin was visualized with Alexa-488 or Alexa-680 phalloidin and nuclei were stained with DAPI (Invtrogen). Slides were viewed with the Leica DMRXA (Leica Microsystems Inc., Bannockburn, IL). Data were collected at 100x and 40x magnification in oil at room temperature. Lenses were 100x oil, numerical aperture 1.4- 0.7, and 40x oil numerical aperture 1.25-0.75. Images were taken using the Coolsnap XQ camera (Photometrics, Tucson, AZ) and processed with Slidebook 5 (Intelligent Imaging Innovations, Inc., Denver, CO). Minimal contrast adjustment was applied equally to experimental and control merged images. Images were sized and annotated using Photoshop (Adobe Systems, Inc., San Jose, CA).

### BMDC phenotype staining

BMDCs were plated in triplicate and primed for 3 hours with 30 ng/ml LPS and then treated with 30 µg/ml purified L1S p60 protein-derived peptide for 4 hours. The cells were then lifted and surface stained for K^b^ (AF6-88.5.5.3), D^b^ (28-14-8), MHC-II (M5/114.15.2), RAE1γ (CX1), RAE1δ (RD-41), MULT-1 (5D10), CD229/Ly9/SLAMF3 (Ly9ab3), Ly-108/SLAMF6 (eBio13G3-19D), CD150/SLAMF1 (9D1), CD84/SLAMF5 (mCD84.7), and CD48/SLAMF2 (HM48-1). All antibodies were from eBioscience (San Diego, CA). Cells were run on a LSRII (BD Biosciences) and 50,000 events were collected. FlowJo software (Tree Star Inc, Ashland, OR) was used to analyze samples.

### Zymography

10 µg each of p60, p60^C389A^, and 0.25 µg of phage autolysin HPL511 were loaded into native 7.5% PAGE gels with .02% heat-killed Lm as PGN substrate. The gels were re-natured in 25 mM Tris ph 7 with 1 mM DTT and 10 mM CaCl_2_, shaking overnight at 37°C. Zymography activity was visualized by staining with 0.01% methylene blue in 0.1%KOH.

### *In vivo* L1S treatment

Female mice between ages 8–10 weeks were treated intraperitonally with 500 µg purified L1S or 10 ng/ml LPS in 300 µl 0.2 M sodium phosphate buffer. For NK cell IFNγ intracellular staining, peritoneal infiltrates were harvested by injecting the peritoneum with 10 ml ice cold PBS with 5 mM EDTA. After light shaking, the fluid was recovered, and cells were stained as described below. Spleens were harvested at 24 into RPMI 1640 (Gibco, Invitrogen). Spleens were treated with 1 mg/ml collagenase in Hank's Buffered Salt Solution (HBSS) plus cations (Invitrogen, Carlsbad, CA) for 30 minutes, mashed through a cell strainer into a single cell suspension and treated with RBC Lysis Buffer (0.15 M NH4Cl, 10 mM KHCO3, 0.1 mM Na_2_EDTA, pH 7.4) and stained as described below.

### Splenocyte and peritoneal lavage staining

Splenocytes and Peritoneal infiltrates were counted and 2×10^6^ cells were incubated in RP-10 media (RPMI 1640, 10% FBS, 1% L-glutamine, 1% Sodium Pyruvate, 1% Penicillin, 1% Streptomycin and 0.1% β-mercaptoethanol) plus GolgiPlug (BD Biosciences, Franklin Lakes, NJ) for 3 hours. Cells were then incubated in anti-CD16/32 (2.4G2 hybridoma supernatant) to block Fc receptors. Surface staining was performed first and included anti-CD3 (clone 145 2C11) and anti-NK1.1 (clone PK136). Cells were then fixed and permeabilzed in a 4% paraformaldehyde and saponin solution and stained with anti-IFNγ (clone XMG1.2) and anti-granzyme B (16G6) (eBioscience, San Diego, CA). Cells were run on a LSRII (BD Biosciences) and 100,000 events were collected. FlowJo software (Tree Star Inc, Ashland, OR) was used to analyze samples. Splenocytes from co-culture experiments were collected 10 hours post-infection, cultured with GolgiPlug (BD Biosciences) for 3 hours, and stained as above.

### Cytotoxicity assays

BMDCs (3×10^4^ per well) were treated or not with 10 ng LPS with or without 10 µg L1S per well for 2 hours. NK-enriched NWNA splenocytes were added to the BMDCs at 2 hours as described in NK-activation and Co-culture. After 21 hours of co-culture, the NWNA splenocytes were collected from co-culture, counted, and added to 5×10^4^ B16F10 mouse melanoma cells (ATCC, Manassas, VA) at Effector∶Target ratios of 1∶10, 1∶10, and 10∶1 based on the estimated number of NK cells in the NWNA splenocytes (5%). The effector and target cells were incubated for 4 hours and cytotoxicity based on LDH release was measured using the Cytotox96 cytotoxicity kit as per manufacturer instructions (Promega, Madison, WI).

### Francisella infection

6–8 week old female mice were pre-treated with 300 µl PBS alone or with 500 ng LPS with or without 500 µg purified L1S, injected i.p. After 24 hours, the mice were infected i.p. with ∼10^4^ LVS strain of *F. tularensis ssp. holarctica* LVS (Ft). Livers and spleens were harvested at 96 hours post Ft infection into 0.02% Nonidet P-40. Livers and spleens were homogenized in a protected fume hood for 1 minute and 2 serial dilutions of homogenate were plated on BHI (Brain and Heart Infusion)(BD Biosciences) agar plates. Plates were incubated at 37°C, 7.5% CO_2_ with humidity for 72 hours and colonies were counted to determine colony forming units per organ. Serum levels of IFNγ were measured by ELISA.

### Statistics

Statistical analysis was performed using Graph Pad Prism 5 (La Jolla, CA). *P* values were assessed using unpaired, two-tailed Student's *t* tests (α = 0.05). In the figures, * denotes *P* values between 0.05 and 0.01, ** denotes *P* values between 0.01 and 0.001, and *** denotes *P* values < or  = 0.001.

### Accession numbers

p60 (NCBI accession ZP_05235088.1), Lm 0394 (NCBI accession ZP_05235264.1).

## Supporting Information

Figure S1 **NK cells alone produce IFNγ in response to p60 stimulation in co-culture.** NWNA splenocytes were stained with NK1.1 and CD3, sorted into NK cells (NK1.1+CD3-),T cells (CD3+NK1.1-), and NK1.1-CD3- populations. Each population, alone or in combination, was co-cultured in triplicate with BMDCs treated with 10 ng LPS and 10 µg purified L1S p60 protein-derived peptide (see Figure 5). IFNγ was measured by ELISA 21 h post-infection. Average ± SEM concentrations of IFNγ produced are shown.
(TIF)

Figure S2 **Contact-dependent NK activation by p60-treated BMDCs can be dissociated from MHC down-regulation, NKG2D ligands, and SLAM family member expression.** BMDCs were plated in triplicate and primed with 30 ng/ml LPS with or without 30 µg/ml purified L1S p60 protein-derived peptide (see Figure 5). The BMDC were then stained for MHC molecules K^b^, D^b^, MHC-II, NKG2D ligands RAE1γ, RAE1δ, and MULT1, and SLAM family members SLAMF1, SLAMF2, SLAMF3, SLAMF5, and SLAMF6. Representative histograms are shown; results represent 2 independent experiments.
(TIF)

Figure S3 **The enzymatic activity of p60 is not required for activation of NK cells.** (A) BMDCs from C57B6 and *RIP2^-/-^* mice were infected in triplicate with LmWT (10403s) or the Δp60 mutant strain. NK-enriched NWNA splenocytes were added 2 hours post-infection, and co-culture supernatant was harvested 21 hours post-infection. Average IFNγ concentration is plotted; error bars represent SEM. (B) 10 µg each of p60, p60^C389A^, and 0.25 µg of phage autolysin HPL511 were loaded into native heat-killed Lm PAGE gels. After renaturation and overnight incubation, zymography activity was visualized by staining with methylene blue. The image was inverted using Photoshop. p60 shows weak PGN hydrolase activity compared to the phage autolysin. p60^C389A^ is catalytically inactive. In native zymography gels, p60 activity appears around 150kD. (D) BMDC were treated with 10 ng LPS, with or without 10 µg detoxified p60 protein, or p60 protein with the C389A catalytic domain mutation. NWNA were added 2 hours post infection, and IFNγ was measured by ELISA 21 hours post infection. Average IFNγ levels +/- SEM are shown. Data are representative of at least three experiments. All treatments were performed in triplicate.
(TIF)

Figure S4 **L1S induces dose-dependent IFNγ production in NK cells** ***in vivo*** **.** Mice were injected i.p. with LPS-associated purified L1S peptide at the doses indicated in 250 µl PBS. After 24 hours splenocytes were stained for CD3, NK1.1, and intracellular IFNγ. Shown are graphical representations of the NK1.1+, CD3- cells that stained positive for IFNγ. n = 2 per dose.
(TIF)

Table S1 Primer Table The primers used to clone transgenic *Listeria* strain constructs and His-tagged proteins are listed including name, purpose, and sequence.
(DOC)
