# Supplementary figures and images for: A LysM and SH3-Domain Containing Region of the Listeria monocytogenes p60 Protein Stimulates Accessory Cells to Promote Activation of Host NK Cells
Source: PLoS Pathog. 2011 Nov 3;7(11):e1002368. doi: 10.1371/journal.ppat.1002368 (PMC3207947; doi:10.1371/journal.ppat.1002368)

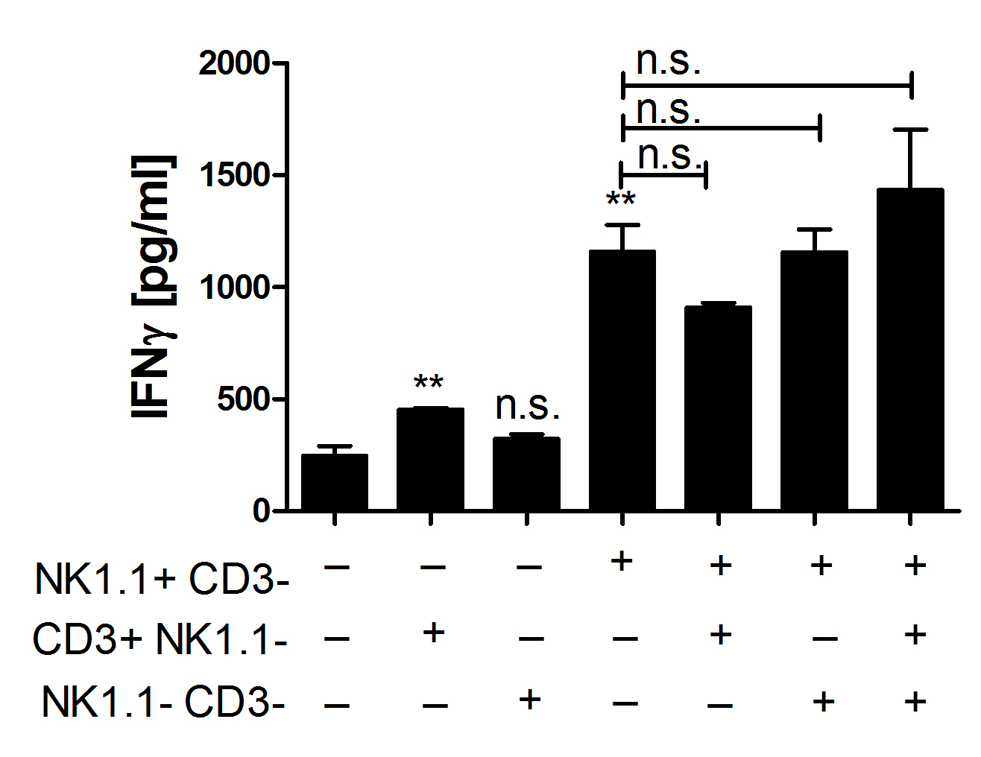

Supplement: Figure S1 — NK cells alone produce IFNγ in response to p60 stimulation in co-culture. NWNA splenocytes were stained with NK1.1 and CD3, sorted into NK cells (NK1.1+CD3-),T cells (CD3+NK1.1-), and NK1.1-CD3- populations. Each population, alone or in combination, was co-cultured in triplicate with BMDCs treated with 10 ng LPS and 10 µg purified L1S p60 protein-derived peptide (see Figure 5). IFNγ was measured by ELISA 21 h post-infection. Average ± SEM concentrations of IFNγ produced are shown. (TIF) [file ppat.1002368.s001.tif]

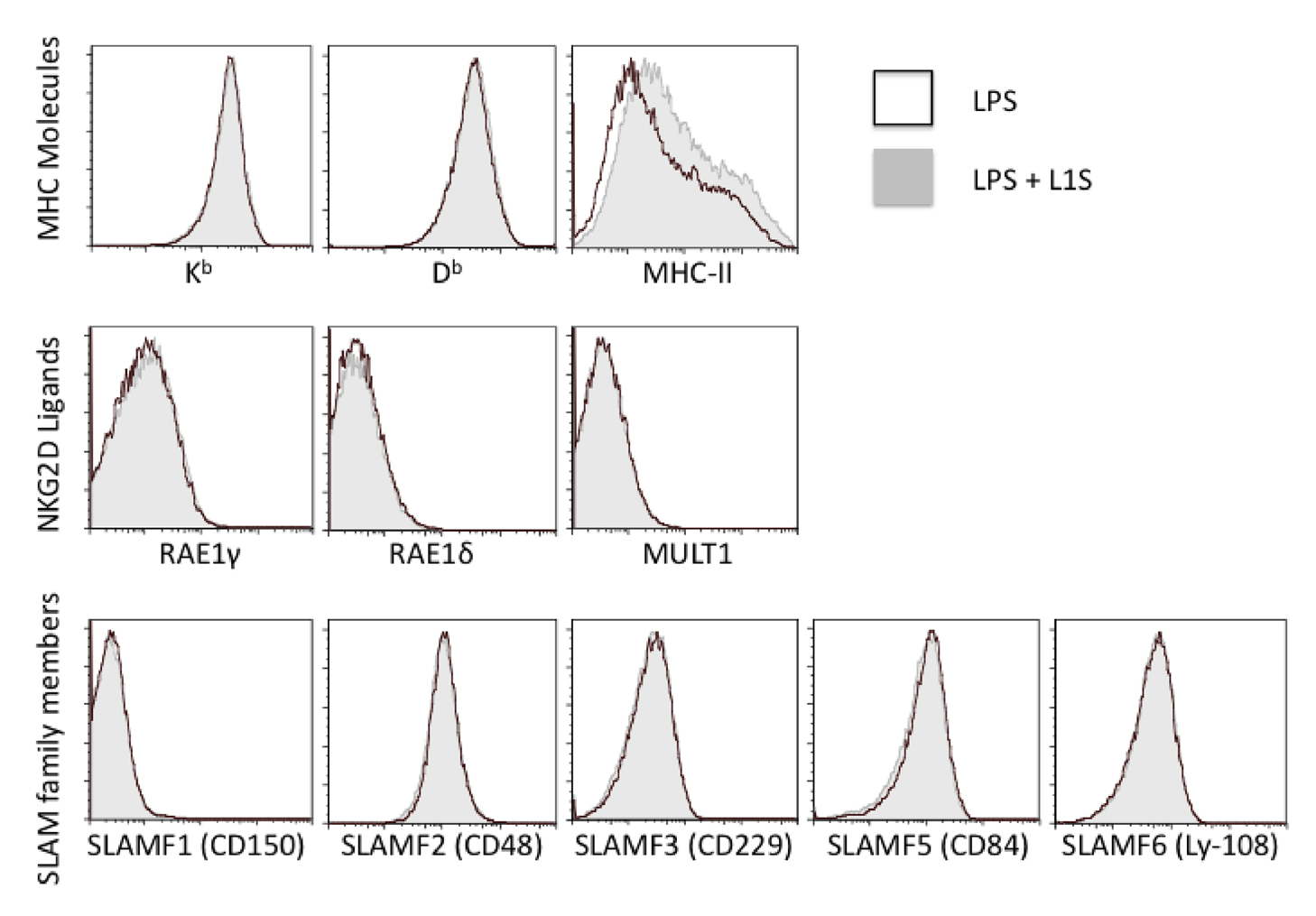

Supplement: Figure S2 — Contact-dependent NK activation by p60-treated BMDCs can be dissociated from MHC down-regulation, NKG2D ligands, and SLAM family member expression. BMDCs were plated in triplicate and primed with 30 ng/ml LPS with or without 30 µg/ml purified L1S p60 protein-derived peptide (see Figure 5). The BMDC were then stained for MHC molecules Kb, Db, MHC-II, NKG2D ligands RAE1γ, RAE1δ, and MULT1, and SLAM family members SLAMF1, SLAMF2, SLAMF3, SLAMF5, and SLAMF6. Representative histograms are shown; results represent 2 independent experiments. (TIF) [file ppat.1002368.s002.tif]

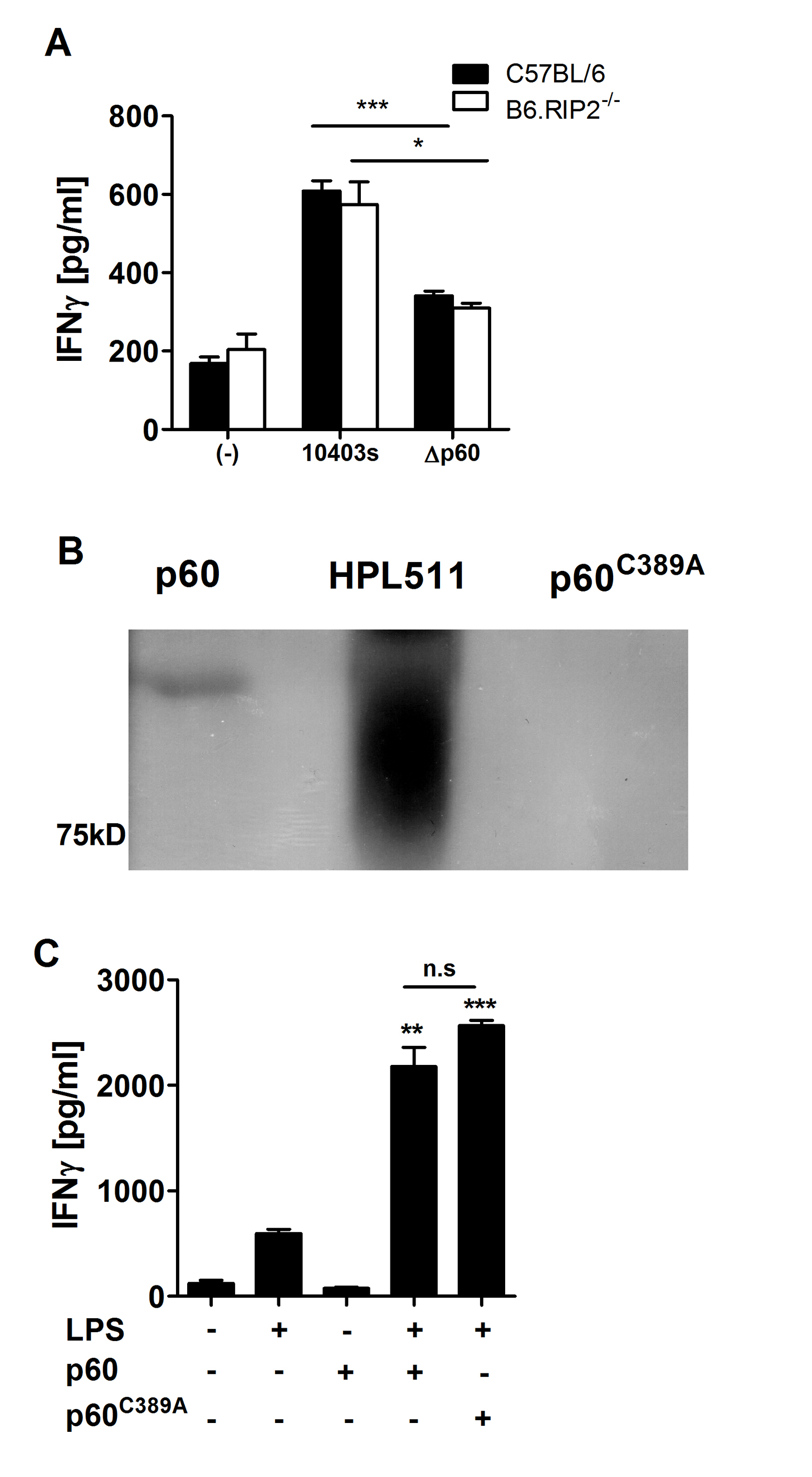

Supplement: Figure S3 — The enzymatic activity of p60 is not required for activation of NK cells. (A) BMDCs from C57B6 and RIP2-/- mice were infected in triplicate with LmWT (10403s) or the Δp60 mutant strain. NK-enriched NWNA splenocytes were added 2 hours post-infection, and co-culture supernatant was harvested 21 hours post-infection. Average IFNγ concentration is plotted; error bars represent SEM. (B) 10 µg each of p60, p60C389A, and 0.25 µg of phage autolysin HPL511 were loaded into native heat-killed Lm PAGE gels. After renaturation and overnight incubation, zymography activity was visualized by staining with methylene blue. The image was inverted using Photoshop. p60 shows weak PGN hydrolase activity compared to the phage autolysin. p60C389A is catalytically inactive. In native zymography gels, p60 activity appears around 150kD. (D) BMDC were treated with 10 ng LPS, with or without 10 µg detoxified p60 protein, or p60 protein with the C389A catalytic domain mutation. NWNA were added 2 hours post infection, and IFNγ was measured by ELISA 21 hours post infection. Average IFNγ levels +/- SEM are shown. Data are representative of at least three experiments. All treatments were performed in triplicate. (TIF) [file ppat.1002368.s003.tif]

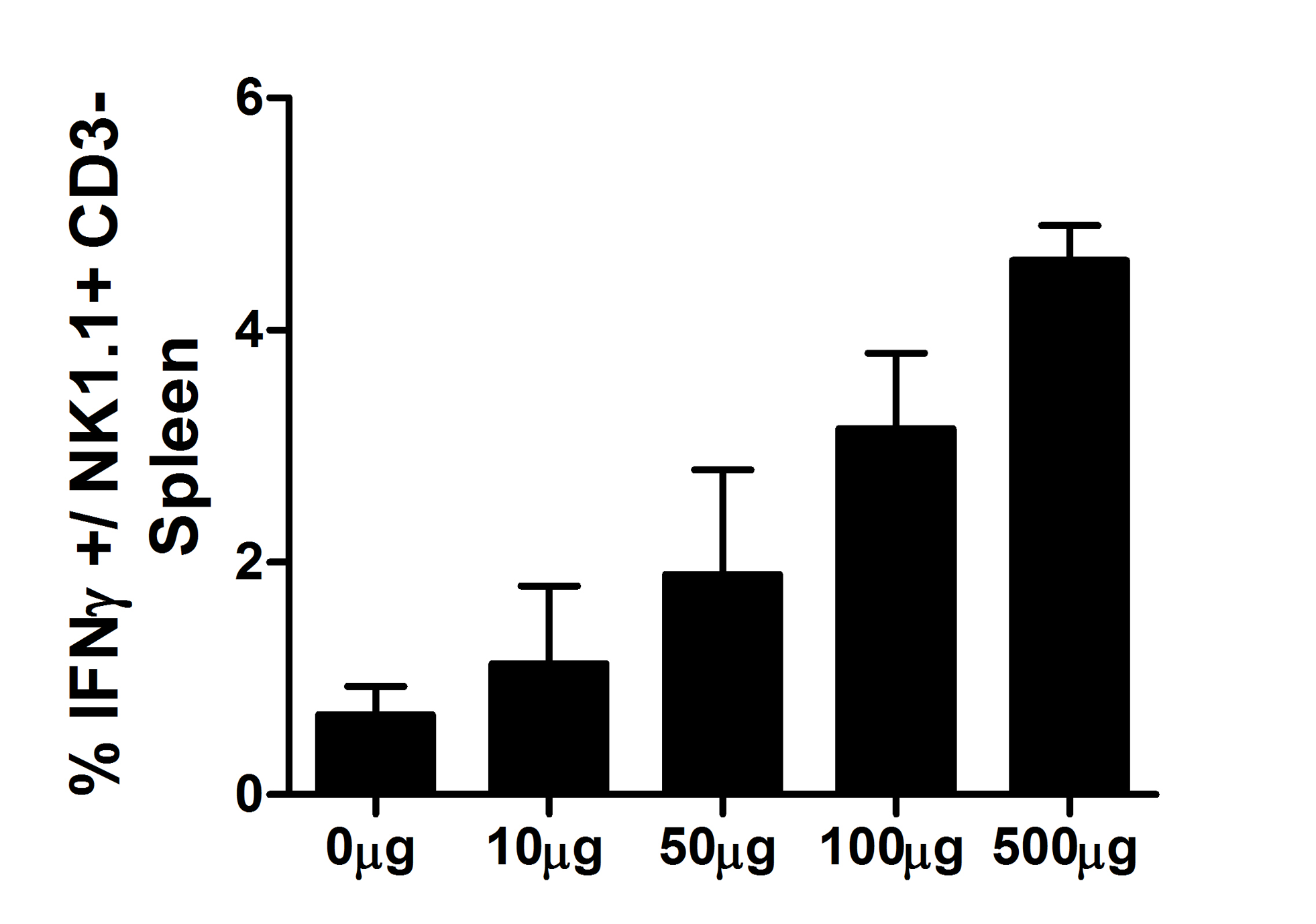

Supplement: Figure S4 — L1S induces dose-dependent IFNγ production in NK cells in vivo . Mice were injected i.p. with LPS-associated purified L1S peptide at the doses indicated in 250 µl PBS. After 24 hours splenocytes were stained for CD3, NK1.1, and intracellular IFNγ. Shown are graphical representations of the NK1.1+, CD3- cells that stained positive for IFNγ. n = 2 per dose. (TIF) [file ppat.1002368.s004.tif]
