# Supplementary material for: A LysM and SH3-Domain Containing Region of the Listeria monocytogenes p60 Protein Stimulates Accessory Cells to Promote Activation of Host NK Cells
Source: PLoS Pathog. 2011 Nov 3;7(11):e1002368. doi: 10.1371/journal.ppat.1002368 (PMC3207947; doi:10.1371/journal.ppat.1002368)
Supplement: Table S1 Primer Table — The primers used to clone transgenic Listeria strain constructs and His-tagged proteins are listed including name, purpose, and sequence. (DOC) [file ppat.1002368.s005.doc]

| **Construct** | **Primer Name** | **Primer Sequence** |
| --- | --- | --- |
| SOE PCR for pIMK2-His-p60 | L3757 | AG **GGATCC** ATG AAA AAG CAA CTA TCG CGG CTA CA |
| **BamHI, *XhoI***, *His tag* | L3758 | ATG ATG ATG *GTG ATG GTG ATG GTG ATG* GCT TGC GGA TGC GAT TGT TGG |
|  | L3759 | AGC *CAT CAC CAT CAC CAT CAC* GTA GTC GAA GCT GGT ACT CTT |
|  | L3760 | AG ***CTCGAG*** TAT ACC GCA CCG AAG CCA ACT AGA TAT TTA CC |
| SOE PCR for pIMK2-∆LysM1p60 | L3757 | AG **GGATCC** ATG AAA AAG CAA CTA TCG CGG CTA CA |
|  | L3928 | CTC TGT TTT TGC GGA TGC GAT TGT TGG AGC AGC AAA |
|  | L3929 | GCA TCC GCA AAA ACA GAG AAA TCT GTT AGC GCA |
|  | L3760 | AG ***CTCGAG*** TAT ACC GCA CCG AAG CCA ACT AGA TAT TTA CC |
| Transfer ∆LDp60 to pPL2 | L3364 | GGGTCGACTCGATCATCATAATTCTGTCTCATT |
| Sal1, **BamH1** | L3365 | CCC**GGATCC**TTATACGCGACCGAAGCCAAC |
| pTrcHis-p60, p60C389A | L3751 | ACTGTAGTAGTCGAAGCTGGTGATACTCTT |
|  | L3752 | AAG AGT ATC ACC AGC TTC GAC TAC TAC AGT |
| p60^C389A^ Site-directed mutagenesis | L3899 | CCAACTCCATTTGATGCTTCTGGTTACACTAAATATG |
|  | L3870 | CATATTTAGTGTAACCAGAAGCATCAAATGTAGTTGG |
| pTrcHis-Np60 | L3751 | ACTGTAGTAGTCGAAGCTGGTGATACTCTT |
|  | L3774 | AGC TTC TGT TGG TGC TTT AGG TGC |
| pTrcHis-Cp60 | L3752 | AAG AGT ATC ACC AGC TTC GAC TAC TAC AGT |
|  | L3773 | ACA AAT GCT AAT AAA ACG AAT ACA AAT ACA AAT ACA AAC AAT ACT AAT ACA |
| pTrcHis-p60L1S | L3751 | ACTGTAGTAGTCGAAGCTGGTGATACTCTT |
|  | L3867 | TTA AGT AGC ATT TTG ATC TAT TAC TGG AGT |
| pTrcHis-Lm0394 | L3860 | ATT TCT TTT TCA GTA CTA TTT TTG CCA ACA ACC |
|  | L3861 | TTA TTT TAA ATT TGC TAC TCG GCC ATA TCC AGC |
